# Anterior Hip Dislocation in a Football Player: A Case Report

**DOI:** 10.1155/2009/363461

**Published:** 2010-02-04

**Authors:** Alexander Schuh, Sylvia Doleschal, Thomas Schmickal

**Affiliations:** ^1^Research Unit Orthopedics and General Surgery, Neumarkt Clinic, 92318 Neumarkt, Germany; ^2^Research Unit, Teaching Hospital of the University of Erlangen-Nurenberg, Nürnberger StraRe 12, 92318 Neumarkt, Germany; ^3^Institution Department of Trauma Surgery, Neumarkt Clinic, 92318 Neumarkt, Germany

## Abstract

Hip dislocations during sporting activities represent only 2%–5% of all hip dislocations. Most hip dislocations in sports can be categorised as “less complicated traumatic hip dislocations” by the Stewart-Milford classification due to the fact that minimal force is involved. The incidence of avascular necrosis of the femoral head greatly increases if the time to reduction is more than six hours. We report the case of a 38-year-old football player who suffered hip dislocation while kicking the ball with the medial aspect of the right foot in an external rotated manner of the right hip. Closed reduction was performed within 2 hours; postoperative follow-up was uneventful. Six months later the patient is out of any complaints; there is no sign of AVN of the femoral head.

## 1. Introduction

Hip dislocations during sporting activities represent only 2%–5% of all hip dislocations [1]. Most hip dislocations are posterior, caused by impaction of the femoral head upon the acetabulum from direct force to the distal femur. Anterior dislocations are less common and of two main types: superior, where the femoral head is displaced into the iliac or pubic region and inferior, where the head lies in the obturator region [2]. Recently Croft et al. [2] published the case of an obturator hip dislocation in a rugby player. We report the case of a 38-year-old football player who suffered an anterior hip dislocation while football playing.

## 2. Case Report

**Figure 1:**
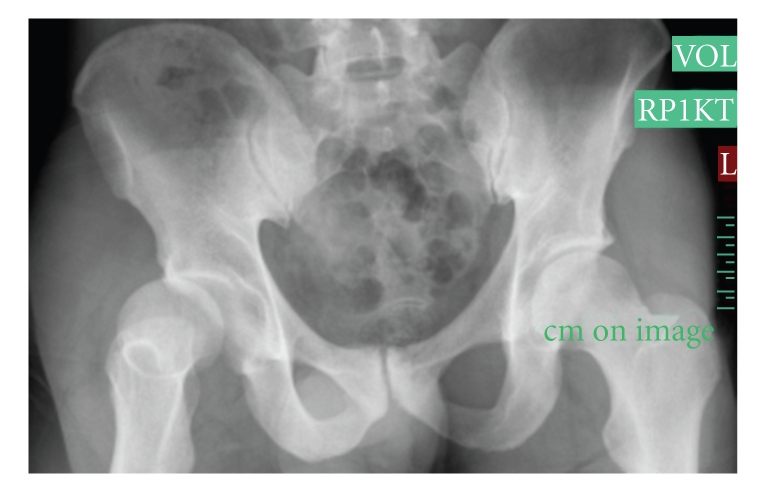
Plain radiograph of the pelvis shows a dislocated right hip.

**Figure 2:**
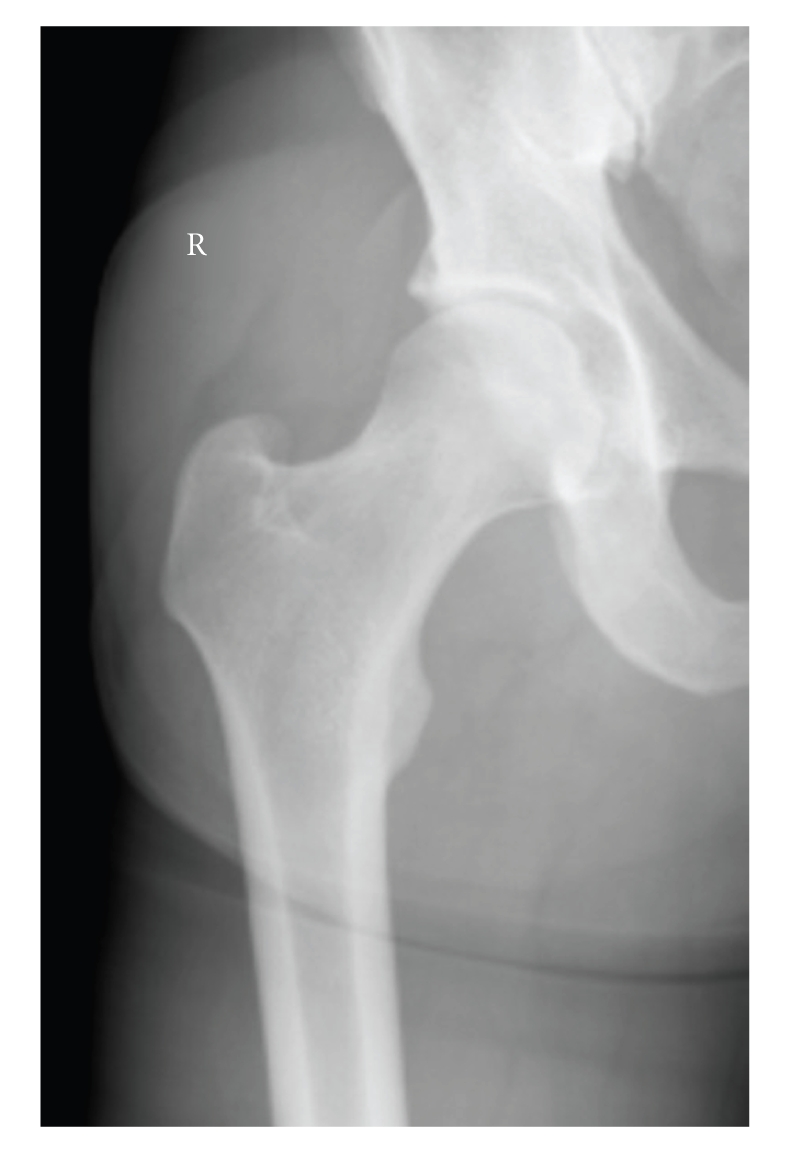
a/p X-ray one week after closed reduction shows a congruent hip joint and no fracture signs.

**Figure 3:**
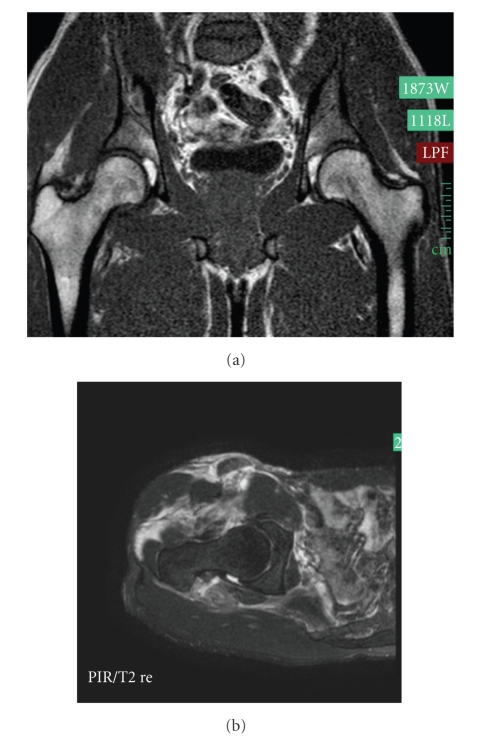
MRI of both hips (a) and transverse cut of the right hip (b) shows no effusion of the congruent right hip, but swelling of the right pelvic muscles.

**Figure 4:**
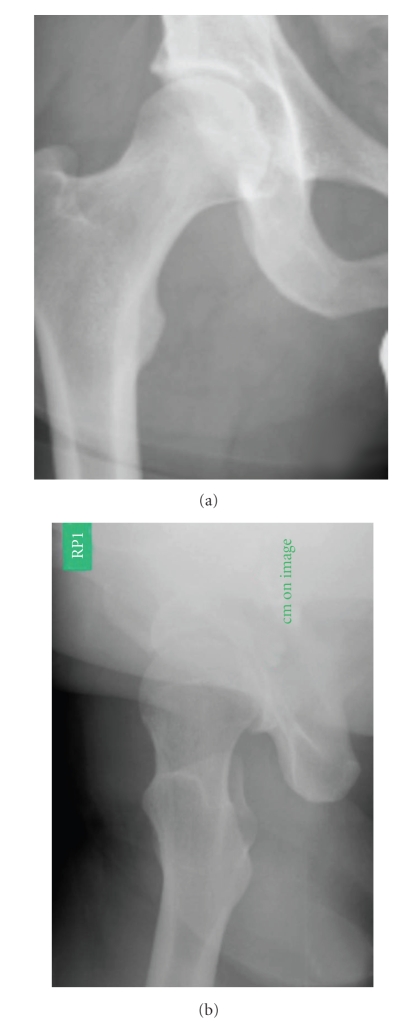
a/p view of the X-ray (a) and lateral view (b) of the right hip 6 months after closed reduction shows a congruent hip joint and no signs of AVN or osteoarthritis.

A 38-year-old man with no medical history suffered an anterior traumatic right hip dislocation while kicking the football with the medial aspect of right the foot in an external rotated manner of the right hip. He then felt a “pop,” and collapsed with an immediate inability to bear weight. There was no contact with an opposing player. Clinically we found an external rotated right leg. Plain radiograph of the pelvis revealed a dislocation of the right hip (Figure 1). A closed reduction using general anesthetic was performed within two hours of the event. The patient lay in a supine position; the pelvis was fixed by an orthopedic surgeon while another orthopedic surgeon performed a continuous axial extension of the right leg till the femoral head was located distal the acetabulum, which was checked by fluoroscopy. At that moment the hip was flexed and internal rotated. This procedure was followed by a snap sound. Fluoroscopy showed the relocated hip. The reposition procedure using the above described technique was out of any difficulties. X-ray taken 1 week after the operation revealed a congruent right hip and no fracture signs (Figure 2). MRI performed 1 week later showed no effusion of the right hip joint but rupture of the iliofemoral ligament and effusion of the pelvic muscles. A labral injury could not be detected, but not ruled out as we did not use an arthro-MRI (Figure 3). The patient had an uneventful postoperative course and was only allowed touch-down weight bearing for 6 weeks. At the six months follow-up, the patient is out of any complaints with a full range of motion of the right hip. X-ray in two planes shows a congruent right hip without signs of AVN or osteoarthritis (Figure 4). As the patient had no complaints and the X-ray showed no signs of AVN, he rejected a proposed MRI. The patient returned to football 6 months after injury.

## 3. Discussion

Hip dislocations (with and without associated acetabular fractures) have been reported in basketball, biking, American football, gymnastics, jogging, rugby, and skiing and soccer [1–15]. Soccer is the world's most popular sport, with over 200 million participants worldwide [1]. Epidemiological studies have shown the incidence of football injuries to be 10–35 per 1000 game hours [5] and it is therefore estimated that every player will have one performance limiting injury a year. The most common injuries are contusions, sprains, and/or strains in the thigh, knee, and ankle. Fractures are relatively uncommon, accounting for 4%–9% of acute injuries [6, 7, 12, 14] and till now only three cases of hip dislocation have been reported [8, 13]. Risk factors of soccer injury have been identified as personal (intrinsic) and/or environmental (extrinsic). Personal risk factors include physical and psychological characteristics of the individual, for example, ligamentous laxity, pre-existing pathological condition of musculoskeletal system, previous injuries, and inadequate rehabilitation. Environmental factors include condition of playing field (uneven, dry, wet), equipment (skin guard, shoes, etc.), rules of the game, and foul play [6]. Goga and Gongal [6] published a case with a fracture dislocation of the hip while running and “hit a high ground” unexpectedly and sustained this injury. In this case the playing field was uneven. Most hip dislocations in sports can be categorised as “less complicated traumatic hip dislocations” by the Stewart and Milford classification [16] due to the fact that minimal force is involved; however, the same prognostic factors that determine the functional outcome in more severe hip injuries apply to the injuries reported here. The incidence of avascular necrosis of the femoral head greatly increases if the time to reduction is more than six hours. The published percentage of avascular necrosis after “less complicated hip dislocations” is 0%–22% [4]. Stiris [13] reported one case of recurrent hip subluxation in a footballer after closed treatment without operative fixation. Neither patient reported here had clinical or radiographic evidence of avascular necrosis at the one year follow-up.

Unfortunately there is no possibility to prevent this rare injury while playing football. Hip dislocation or fracture dislocation is extremely uncommon in football; however, the potentially serious long-term sequelae the team doctor should be aware of this injury. Furthermore, our case reported in this paper illustrate the importance of early reduction to produce a stable and congruent joint in order to decrease the risk of subsequent osteonecrosis as reduction may relieve tension across the femoral and circumflex vessels.

## References

[1] Giza E, Mithöfer K, Matthews H, Vrahas M (2004). Hip fracture-dislocation in football: a report of two cases and review of the literature. *British Journal of Sports Medicine*.

[2] Croft SJ, Brenchley J, Badhe SP, Cresswell TR (2006). An unusual rugby injury. *Emergency Medicine Journal*.

[3] Brooks JHM, Fuller CW, Kemp SPT, Reddin DB (2005). Epidemiology of injuries in English professional rugby union—part 2 training injuries. *British Journal of Sports Medicine*.

[4] Chudik SC, Allen AA, Lopez V, Warren RF (2002). Hip dislocations in athletes. *Sports Medicine and Arthroscopy Review*.

[5] Dahan R (1997). Rehabilitation of muscle-tendon injuries to the hip, pelvis, and groin areas. *Sports Medicine and Arthroscopy Review*.

[6] Goga IE, Gongal P (2003). Severe soccer injuries in amateurs. *British Journal of Sports Medicine*.

[7] Hawkins RD, Hulse MA, Wilkinson C, Hodson A, Gibson M (2001). The association football medical research programme: an audit of injuries in professional football. *British Journal of Sports Medicine*.

[8] Lamke LO (1970). Traumatic dislocations of the hip. *Acta Orthopaedica Scandinavica*.

[9] Mohanty K, Gupta SK, Langston A (2000). Posterior dislocation of hip in adolescents attributable to casual rugby. *Journal of Accident and Emergency Medicine*.

[10] O’Leary C, Doyle J, Fenelon G, Ward F (1987). Traumatic dislocation of the hip in Rugby Union football. *Irish Medical Journal*.

[11] Pallia CS, Scott RE, Chao DJ (2002). Traumatic hip dislocation in athletes. *Current Sports Medicine Reports*.

[12] Söderman K, Adolphson J, Lorentzon R, Alfredson H (2001). Injuries in adolescent female players in European football: a prospective study over one outdoor soccer season. *Scandinavian Journal of Medicine and Science in Sports*.

[13] Stiris MG (2000). MR imaging after sports-induced HIP dislocations: report of three cases. *Acta Radiologica*.

[14] Sullivan JA, Gross RH, Grana WA, Garcia-Moral CA (1980). Evaluation of injuries in youth soccer. *American Journal of Sports Medicine*.

[15] Yates C, Bandy WD, Blasier RD (2008). Traumatic dislocation of the hip in a high school football player. *Physical Therapy*.

[16] Stewart M, Milford L (1954). Fracture-dislocation of the hip: an end-result study. *Journal of Bone and Joint Surgery*.

